# A comparison of European, Polish, Slovenian and British EQ-5D-3L value sets using a Hungarian sample of 18 chronic diseases

**DOI:** 10.1007/s10198-019-01069-8

**Published:** 2019-05-18

**Authors:** Zsombor Zrubka, Zsuzsanna Beretzky, Zoltán Hermann, Valentin Brodszky, László Gulácsi, Fanni Rencz, Petra Baji, Dominik Golicki, Valentina Prevolnik-Rupel, Márta Péntek

**Affiliations:** 1 Department of Health Economics, Corvinus University of Budapest, Fővám tér 8., Budapest, 1093 Hungary; 2 Doctoral School of Business and Management, Corvinus University of Budapest, Fővám tér 8., Budapest, 1093 Hungary; 3 Centre for Economic and Regional Studies, Hungarian Academy of Sciences, Tóth Kálmán u. 4, Budapest, 1097 Hungary; 4 Centre of Labour Economics, Corvinus University of Budapest, Fővám tér 8., Budapest, 1093 Hungary; 5 Premium Postdoctoral Research Program, Hungarian Academy of Sciences, Nádor u. 7, Budapest, 1051 Hungary; 6 Department of Clinical and Experimental Pharmacology, Medical University of Warsaw, Banacha 1B, Warsaw, 02-097 Poland; 7 Institute for Economic Research, Kardeljeva ploščad 17, 1109 Ljubljana, Slovenia

**Keywords:** EQ-5D-3L, Value set, Chronic disease, Quality of life, Disease burden, Hungary, Slovenia, Poland, I10

## Abstract

**Background:**

In the Central and Eastern European region, the British EQ-5D-3L value set is used commonly in quality of life (QoL) studies. Only Poland and Slovenia have country-specific weights. Our study aimed to investigate the impact of value set choice on the evaluation of 18 chronic conditions in Hungary.

**Methods:**

Patients’ EQ-5D-3L index scores were calculated using the VAS-based Slovenian and European and the time-trade-off-based Polish and British value sets. We performed pairwise comparisons of mean index values by dimensions, diagnoses and age groups. We evaluated disease burden by comparing index values matched by age and gender in each condition with those of the general population of the CEE region in all four value sets.

**Results:**

Altogether, 2421 patients (55% female) were included in our sample with the average age of 55.87 years (SD = 17.75). The average Slovenian, European, Polish and British EQ-5D-3L scores were 0.598 (SD = 0.279), 0.661 (SD = 0.257), 0.770 (SD = 0.261) and 0.644 (SD = 0.279), respectively. We found highly significant differences in most diagnoses, with the greatest difference between the Polish and Slovenian index values in Parkinson’s disease (0.265). Systematic pairwise comparison across all conditions and value sets revealed greatest differences between the time-trade-off (TTO) and VAS-based value sets as well as varying sensitivity of the disease burden evaluations of chronic disease conditions to the choice of value sets.

**Conclusions:**

Our results suggest that the choice of value set largely influences the health state utility results in chronic diseases, and might have a significant impact on health policy decisions.

## Introduction

The burden of chronic diseases is ever increasing as these conditions are the main causes of poor health, disability, and account for most the health care expenditures nowadays [1]. Chronic diseases are often lifelong conditions, which require constant treatment and cause significant burden not only on the individual but also on a societal level. The introduction of new therapies for the treatment of chronic conditions requires knowledge on the potential health gains in terms of life years gained or improved quality of life. Measuring health-related quality of life (HRQoL) in chronic diseases could aid the evaluation of healthcare interventions’ effectiveness and provide information on the potential health gain that can be achieved. Information on potential health gains contributes to the better allocation of resources and provides input to decision-making.

Evidence on patient’s health-related quality of life can be obtained using patient-reported outcome measures, some of which are generic, meaning they cover a more general spectrum of health problems and are designed to be applicable for various health conditions [2]. The EQ-5D-3L questionnaire is a generic measure of health, which is widely used in different countries in a variety of clinical areas. The EQ-5D-3L questionnaire can be used to derive Health Utility Index scores [3]. The utility values that are attached to the health states described by the questionnaire can be used in health economic analysis to quantify the possible health gains expressed in quality-adjusted life years (QALYs) [4].

For the valuation of the EQ-5D-3L health profiles, different value sets are applicable. However, not all countries have their own value set: in the Central and Eastern European (CEE) regions, only Slovenia and Poland have country-specific value sets. In health economic analyses as well as the assessment of the health status of the general population, in other countries of the CEE region, usually, the British TTO value set is used [5–8]. The European value set was derived from six countries [9], to be used in multinational studies in Europe. However, it is not frequently used in the CEE region. [8].

Previous studies indicate that differences are present between results calculated with different value sets and that it may be attributed to societal and cultural differences. The relative importance of the problems reported in the five EQ-5D dimensions differs by value sets [10], and certain conditions differ largely in the frequency of problems reported in the various EQ-5D dimensions. Therefore, differences in the country-specific value sets may lead to significantly different EQ-5D index scores and thus differences in utility values among different disease conditions. As the utilities are often used in health economic analyses, the use of different value sets may lead to disparities and significant differences in health priority setting and decision-making. Comparative analyses have been conducted to determine how the choice of value sets might affect the assessment of population health [11], QALY calculations in patient samples [12] or the assessment of health state transitions [13]. Furthermore, the measurement properties of EQ-5D-5L and 3L value sets from several countries have been compared in eight disease groups. [14] However, we were not aware of studies exploring the systematic differences between value sets across multiple disease conditions and their potential impact on disease burden evaluations and health priority setting.

We aimed to compare the Slovenian, Polish, British and European EQ-5D-3L value sets, which are most commonly used or are potentially applicable for health economic evaluations in the CEE region. Our study was based on the comparative analysis of patient-level data from cross-sectional surveys conducted in Hungary among patient populations in 18 different chronic conditions. We explored the differences of the EQ-5D-3L index scores calculated with the four value sets by diagnosis, age group and disease severity. Furthermore, we analysed the potential impact of the choice of value sets on health priority setting by comparing the disease burden evaluations across different conditions using different value sets.

## Methods

### EQ-5D-3L

The EQ-5D-3L questionnaire is a health status measure that consists of two parts, a descriptive system and the EQ VAS. The descriptive system focuses on five dimensions of health: mobility (MO), self-care (SC), usual activities (UA), pain/discomfort (PD) and anxiety/depression (AD). In each dimension, there are three response categories (1, 2 or 3) representing: no problems—1, moderate problems (some problems in MO, SC and UA; moderate PD and AD)—2; severe problems (unable MO, SC and UA, extreme PD and AD)—3. Respondents are asked to indicate for each dimension the level of problem that best describes their current health status [5, 15]. The responses (1, 2 or 3) for the five dimensions can be combined into a five-digit number that describes the respondent’s health state (e.g. no problem in any of the five dimensions can be described as 11111; some problems in Mobility but no problem in the other four dimensions is presented as 21111). The descriptive system can define 243 different health states. To each health state, an EQ-5D-3L index score can be attached according to a particular set of preference weights, also called value set. The EQ-5D-3L index score (value) reflects the relative utility (desirability) of the health state and can be used for the calculation of quality-adjusted life years (QALY) in cost-utility analyses [4]. The second part of the questionnaire is a visual analogue scale (EQ VAS) ranging from 0 (representing the worst imaginable health) to 100 (best imaginable health state) on which respondents rate their current health state.

### EQ-5D-3L value sets

EQ-5D-3L value sets have been developed in various countries based on preferences of the general population using direct methods such as time-trade-off (TTO) and EQ-5D visual analogue scale (EQ-VAS, hereinafter VAS) or both [16]. In the TTO exercise, respondents have to decide between two alternative outcomes. Generally, the question refers to whether they would be willing to exchange a certain life expectancy to a shorter one lived in perfect health [17]. In the VAS-based valuation, respondents have to locate the different health states as well as death on the 0–100 VAS, where 0 is the worst and 100 is the best imaginable health state. The differences between the positions of the health states marked on the VAS should correspond to the differences in preference as perceived by the respondent.

In our study, we compared four value sets. The British valuation study was conducted in 1993. From 6080 randomly selected addresses, 2997 respondents provided complete data for evaluation. The value set was determined by the valuation of 42 health states in a face-to-face TTO exercise in addition to “full health” (11111) and “death”. Each respondent valued 13 health states. [7]. A VAS-based European value set was published in 2003 [9]. It was derived from 11 surveys carried out between 1991 and 1999 in Finland (one study), Germany (three studies), The Netherlands (one study), Spain (three studies), Sweden (two studies) and the UK (two studies), with a total of 6870 respondents [18–28]. The preference weights were estimated from the valuations of 44 EQ-5D health states. In the individual studies, various combinations of the 44 health states were valued. However, most studies valued a core set of 13 health states in addition to full health and death, and each of the 44 states were valued in at least two studies. [9]. The Polish valuation study was performed in 2008 on a sample of 321 visitors of inpatients in eight hospitals using quotas to ensure a representative sample of the adult population of Poland. The TTO exercise was conducted during face-to-face interviews, and 45 health states were valued in addition to 11111 and “dead”. Each respondent valued 25 health states. [29]. The Slovenian value set was determined in 2000 in a postal survey. The survey involved 3000 adults randomly selected from the general population. After checking logical consistency, answers returned by 370 individuals (12.3%) were used in the valuation exercise. In addition to “dead” and “unconscious”, each respondent valued the same 14 health states using the VAS [30]. The EQ-5D-3L index score range of the four value sets are as follows: Slovenian − 0.225 to 1; European − 0.074 to 1; Polish − 0.523 to 1; British − 0.594 to 1.

### Sample

This current study is a secondary analysis of 18 previous surveys conducted by the Department of Health Economics of the Corvinus University of Budapest in the past 14 years [8, 31–48]. We selected those studies which used the validated Hungarian version of the EQ-5D-3L questionnaire for the assessment of patients with chronic conditions. We only included those patients in our analysis, who had answers in all five EQ-5D-3L dimensions; hence EQ-5D-3L index scores could be calculated using the four different value sets. The 18 datasets were combined into a pooled sample.

### Statistical analysis

We applied descriptive methods and graphical representation of key findings. As sample sizes varied substantially across the datasets (min: *N* = 61, max: *N* = 249), analytical weights were constructed to make the pooled dataset a balanced sample of the 18 diseases. The sum of weights was set to 100 by each condition. We calculated weighted mean and percentage values when reporting characteristics of the pooled sample totals. We compared the four value sets by (1) EQ-5D-3L dimensions, (2) by diagnosis, (3) by respondents’ subjective health assessment (EQ VAS) and (4) by age group, according to the following. (1) When comparing value sets by EQ-5D-3L dimensions, we graphically represented indices of health states with moderate and severe levels of isolated problems in each dimension (e.g. 21111, 31111, etc.), as well as the combinations of moderate and severe problems (21122, 22222, 32233, 33333) against full health (11111). This comparison allows us to take into account the full disutility arising from the severity of problems and the dimension-specific preferences [10]. (2) Although the distribution of index values was not normal, the sample size was sufficiently large to allow the comparison of diagnosis subgroups using two-sided paired *t* tests [49]. (3) To assess the differences between value sets depending on disease severity, we plotted the four value sets against respondents’ subjective health assessment. We obtained EQ VAS scores from each patient in addition to the EQ-5D-3L health profiles. To balance frequency peaks at round EQ VAS values, yet retain granularity, we divided patients into 36 quantiles based on EQ VAS scores, and represented visually mean index values of the four value sets in each EQ VAS quantile. (4) When comparing value sets across age groups, we applied a weighted OLS regression model with standard errors clustered at the individual patient level.

Finally, for each value set, we calculated disease burden (DB) scores and the sensitivity of DB evaluations to the choice of value set. DB was defined as the difference between the average health status of the patient group and health state of the general population comparable by age and gender. For DB score calculations, we used the population norm values estimated from a joint database of Polish, Slovenian and Hungarian population studies (*n* = 6926), by calibrating to the 2011 European population census by age, gender and education [50]. We expressed DB scores as disutilities. For instance, a 0.2-point DB score over a year represents 0.2 QALY loss compared to the comparable general population.

From the 18 samples, we modelled the effect of the choice of the value set on DB evaluations. We calculated a sensitivity index according to the following procedure. First, we performed all pairwise DB comparisons for each condition using the four value sets. Each *i* condition was compared to the other 17 *j* conditions (*i* ≠ *j*). In each comparison with value set *k* (Slovenian, European, Polish or British), the difference between DB_*i*_ and DB_*j*_ (ΔDB_*ijk*_) could be significantly positive (+), non-significant (0) or significantly negative (−). Second, we evaluated how consistent ΔDB_*ij1*···_ΔDB_*ijk*_ were across the *k* value sets. In case of consistent difference, the pattern was identical for all four value sets (++++ , 0000 or −−−−). In case of inconsistent difference, the outcome was diverse (e.g. 0 + 00, or 00−−). When comparing value set pairs, then the pattern of difference was assessed on two ΔDB_*ij*_ pairs (*k *= 2). The pattern of consistent difference was ++, 00 or −−, while examples of inconsistent difference were 0 + , −0 or +−, etc. Finally, the sensitivity index was expressed as the proportion of diverse outcomes among all pairwise comparisons. We calculated sensitivity indices by comparing the four value sets for each diagnosis as well as for the total sample. These sensitivity indices can be interpreted as a general measure of how sensitive the DB evaluations are to the choice of a value set. For each diagnosis, the general sensitivity index calculation was based on 17 diagnosis pairs and four value sets (DB_*ij*_*n *= 17, *k *= 4). For the sample total, the general sensitivity index calculation was based on 153 diagnosis pairs and four value sets (ΔDB_*ij*_*n* = 18*17/2, *k *= 4). The pairwise sensitivity indices express for each diagnosis how sensitive the DB evaluations are to the choice between two particular value sets (DB_*ij*_*n *= 17, *k *= 2), as well as how sensitive the sample total is to the choice between two particular value sets (DB_*ij*_*n *= 18*17/2, *k *= 2). Table 3 summarises the sensitivity indices denoting the calculation methods. When interpreting the results, greater sensitivity index values represent a greater share of inconsistent comparisons or greater sensitivity to the value set choice.

## Results

### Sample characteristics

The 18 chronic conditions belonged to nine different ICD-10 (International Statistical Classification of Diseases and Related Health Problems 10th Revision) groups. The total sample included 2421 patients with psoriatic arthritis (PsA) [31], age-related macular degeneration (AMD) [32], attention-deficit/hyperactivity disorder (ADHD) [33], dementia [34], diabetes mellitus (DM) [35], endometriosis (ENDO) [36], epilepsy [37], bladder cancer (BC) [38], benign prostatic hyperplasia, (BPH) [39], osteoporosis (OP) [8], peripheral arterial occlusive disease (PAOD) [40], Parkinson’s disease (PD) [41], psoriasis (PSO) [42, 43], rheumatoid arthritis (RA) [44], overactive bladder (OAB) [45], systemic sclerosis (SSc) [46], multiple sclerosis (MS) [47] and schizophrenia (SCZ) [48]. The mean age of the patients was 55.87 years (SD = 17.75). More than half of patients were older than 70 years in dementia, AMD, BPH and PAOD. More than half of the patients were female (*n* = 1356, 58.6%) and it is worthy of note that some studies involved exclusively female (endometriosis, osteoporosis, OAB) or male (BPH) patients. The mean disease duration in our sample was 8.75 (SD = 8.95) years, with outstandingly high average disease duration in patients with psoriasis and epilepsy (Table 1).

**Table 1:** Main characteristics of the patient samples

ICD-10 category/diagnosis	Number of patients (*n*)	Age, mean (SD)	Female, *n* (%)	Disease duration (year), mean (SD)	EQ VAS mean (SD)
Diseases of the musculoskeletal system and connective tissue					
RA	249	55.38 (12.32)	214 (86.3%)	9.15 (9.33)	51.59 (19.90)
PsA	177	49.89 (12.76)	101 (57.1%)	9.30 (9.24)	55.02 (19.87)
SSc	80	57.39 (9.60)	72 (90.0%)	7.16 (6.63)	56.25 (18.73)
OP	207	69.57 (8.93)	207 (100%)	7.49 (5.60)	59.20 (17.19)
Diseases of the nervous system					
Epilepsy	96	36.16 (12.12)	56 (58.3%)	15.38 (11.55)	73.84 (16.16)
MS	68	37.96 (9.08)	48 (70.6%)	7.02 (5.90)	64.74 (22.18)
PD	99	62.67 (11.32)	31 (33.0%)	8.08 (5.59)	59.47 (18.28)
Mental, behavioural and neurodevelopmental disorders					
Dementia	86	77.61 (8.60)	51 (60.0%)	NA	48.59 (23.88)
ADHD	75	30.44 (10.49)	17 (22.7%)	NA	69.45 (19.43)
SCZ	78	44.24 (13.05)	36 (46.2%)	NA	60.01 (24.71)
Diseases of the genitourinary system					
ENDO	79	32.67 (4.80)	79 (100%)	7.68 (6.33)	NA
BPH	237	70.38 (8.18)	0 (0.0%)	5.53 (4.79)	68.44 (15.63)
OAB	61	57.72 (11.56)	61 (100.0%)	NA	62.80 (18.80)
Diseases of the skin and subcutaneous tissue					
PSO	192	50.49 (12.79)	61 (31.8%)	21.66 (11.77)	64.49 (21.52)
Neoplasms					
BC	148	66.24 (9.61)	50 (33.8%)	3.56 (3.78)	67.82 (19.35)
Endocrine, nutritional and metabolic diseases					
DM	264	61.31 (10.98)	151 (57.2%)	NA	62.12 (19.95)
Diseases of the circulatory system					
PAOD	103	70.00 (10.21)	45 (43.7%)	NA	45.75 (16.28)
Diseases of the eye and adnexa					
AMD	122	75.16 (7.88)	76 (62.3%)	2.94 (2.54)	58.59 (16.43)
Total^a^	2421	55.87 (17.75)	1356 (58.6%)	8.75 (8.95)	60.46 (20.74)

*ADHD* attention-deficit/hyperactivity disorder, *AMD* age-related macular degeneration, *BC* bladder cancer, *BPH* benign prostatic hyperplasia, *DM* diabetes mellitus, *ENDO* endometriosis, *NA* not available, *MS* multiple sclerosis, *OAB* overactive bladder, *OP* osteoporosis, *PAOD* peripheral arterial occlusive disease, *PsA* psoriatic arthritis, *PSO* psoriasis, *RA* rheumatoid arthritis, *SCZ* schizophrenia, *SSc* systemic sclerosis

^a^Weighted mean and percentage values using analytical weights summing up to 100 in each disease

### Problems reported in the five EQ-5D-3L dimensions

In our sample, 519 patients (20.7%) did not report any problem in any of the five EQ-5D-3L dimensions, while 420 (16.1%) reported problems (of any level) in all the five dimensions. Severe problems in at least one dimension were reported by 419 patients (17.6%), while 2002 patients (82.4%) did not report the severe problem in any of the five dimensions.

**Fig. 1:**
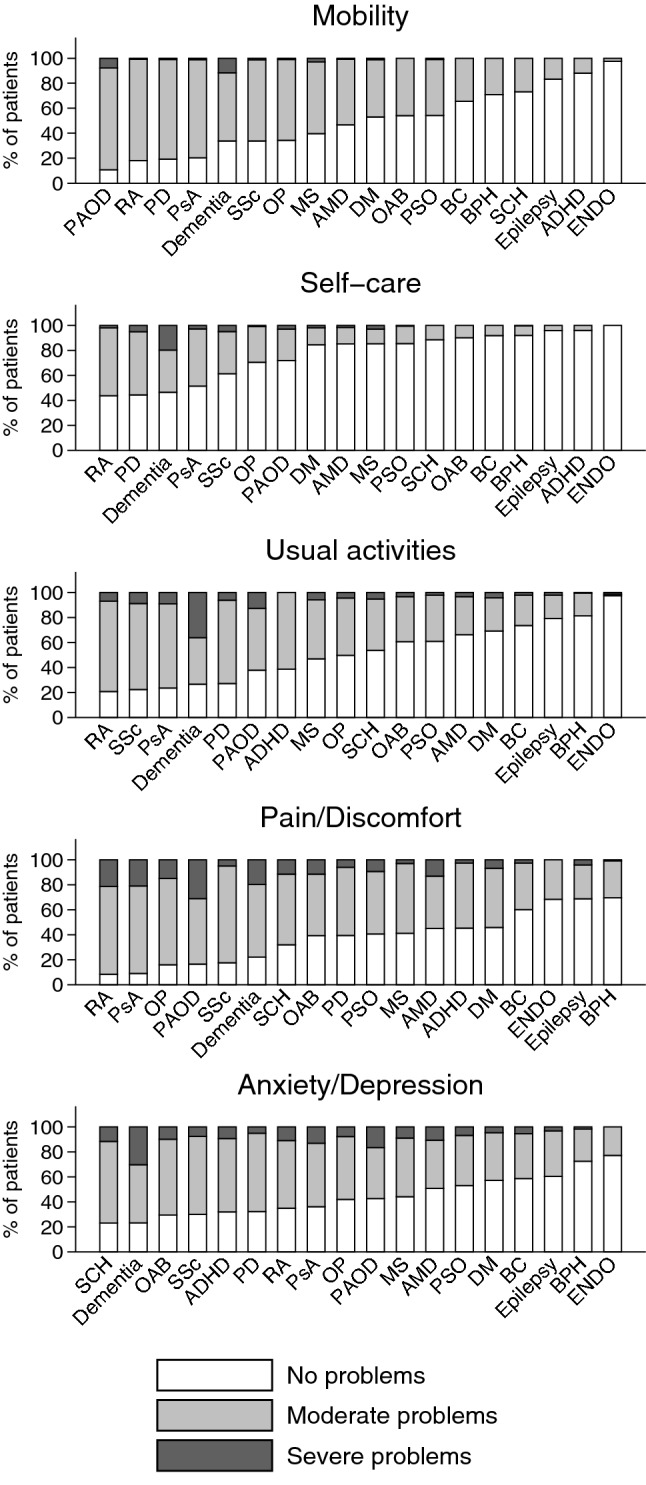
Problems reported in the five EQ-5D-3L dimensions by diagnosis. *ADHD* attention-deficit/hyperactivity disorder, *AMD* age-related macular degeneration, *BC* bladder cancer, *BPH* benign prostatic hyperplasia, *DM* diabetes mellitus, *ENDO* endometriosis, *MS* multiple sclerosis, *OAB* overactive bladder, *OP* osteoporosis, *PAOD* peripheral arterial occlusive disease, *PsA* psoriatic arthritis, *PSO* psoriasis, *RA* rheumatoid arthritis, *SCZ* schizophrenia, *SSc* systemic sclerosis

The distribution of patients by problem levels and diagnoses across the five EQ-5D-3L dimensions are presented in Fig. 1. Any problems were reported most frequently in dementia (96.5%), RA (95.2%), PAOD (95.2%) and AP (94.4%), while least frequently in endometriosis (44.3%), epilepsy (52.1%) and BPH (53.2%). Considering the total sample, the less affected health dimension was self-care with moderate and severe problems reported by 20.5% and 2.6% of respondents, respectively. Most problems were indicated in the pain/discomfort dimension followed by anxiety/depression, with moderate problems reported by 51.7% and 46.5%, and severe problems reported by 10.3% and 9.0%, respectively.

The percentage of patients reporting any problems in mobility, self-care, usual activities, pain/discomfort and anxiety/depression was greatest in PAOD (89.3%), RA (56.2%), RA (20.9%), RA (8.4%) and SCZ (76.9%), respectively. Patients with dementia reported severe problems most frequently in the mobility (11.3%), self-care (19.7%), usual activities (36.1%) and anxiety/depression (30.2%) dimensions, while PAOD patients reported most frequently severe problems in the pain/discomfort dimension (31.1%) (Fig. 1).

### Comparison of value sets by EQ-5D-3L dimensions

**Fig. 2:**
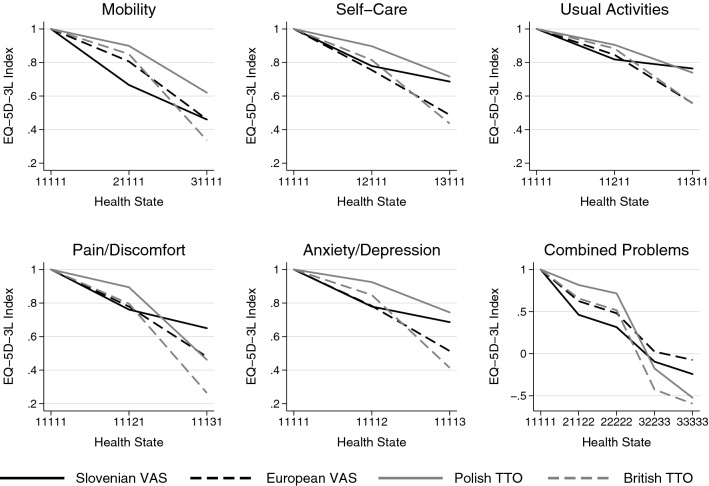
EQ-5D-3L index scores for selected health states by the four different value sets

Figure 2 illustrates the relative importance of the EQ-5D-3L dimensions in the four value sets. Except for severe problems in the usual activities and pain/discomfort dimensions, the polish value set provided the highest values for health states with moderate or severe problems in a single dimension. For moderate problems, the Slovenian, while for severe ones the British value set provided the lowest values. The disutility of moderate problems compared to full health tended to be relatively small in the Polish and British value sets, followed by a steep decline between moderate and severe problem levels. However, in the Slovenian value set, the disutility of moderate problems tended to be greater, followed by a moderate decline at severe problems. The differences between full health and moderate problems and between moderate and severe problems were similar in the European value set. The index values of health states with combined problems showed that compared to the TTO-based value sets the VAS-based Slovenian and European value sets tended to have lower index values in milder health states, while the UK and Polish value sets provided the lowest values for the combination of severe problems (33333). Among all 243 EQ-5D-3L profiles, “worse than dead” health states with negative utility values were most prevalent in the British value set (35%), followed by the Polish (13%), the Slovenian (9%) and the European one (2%).

Considering severe problems, the Polish and British value sets provided the largest decrease of health state value in the pain/discomfort dimension. In contrast, severe problems in the mobility dimension had the highest negative impact on the EQ-5D-3L index value in the European and the Slovenian value sets. Based on these findings, we expected that both the overall severity and the main problem domains of certain conditions would contribute to the utility differences demonstrated between the four value sets.

### Comparison of value sets by diagnosis

To conduct our analysis by diagnosis, we calculated the EQ-5D-3L index scores (mean, standard deviation) with all the four value sets for each diagnosis. The weighted mean EQ-5D-3L index scores in our total sample were, respectively, 0.598 (SD = 0.279), 0.661 (SD = 0.257), 0.770 (SD = 0.261) and 0.644 (SD = 0.334) with the Slovenian, European, Polish and British value sets. All pairwise value set comparisons were significant (*p* < 0.001). Patients with endometriosis had the highest, while dementia patients had the lowest mean EQ-5D-3L scores in all four value sets. The most significant difference between any two value sets was found in PD: the difference between the Slovenian and Polish index values was 0.265. The pairwise comparisons of the four value sets by two-sided *t* tests showed highly significant differences in most diagnoses. In SCZ, the British value set did not differ significantly from the Slovenian and European value sets. In other diagnoses, five or six pairwise value set comparisons were significantly different. The British and European value sets did not differ significantly in 10, and the British and Slovenian in 2 out of the 18 diagnoses. All other value set comparisons showed significant differences in all diagnoses (Table 2).

**Table 2:** EQ-5D-3L index scores by diagnosis

Patients, ICD-10 categories and diagnoses	EQ-5D-3L index score, mean (SD)				Two-sided paired *t* test *p* values					
	SI	EU	UK	PL	SI-EU	SI-PL	SI-UK	PL-UK	PL-EU	UK-EU
Diseases of the musculoskeletal system and connective tissue										
RA	0.411 (0.217)	0.506 (0.235)	0.464 (0.334)	0.646 (0.270)	< 0.001	< 0.001	< 0.001	< 0.001	< 0.001	< 0.001
PsA	0.423 (0.230)	0.513 (0.244)	0.467 (0.347)	0.645 (0.288)	< 0.001	< 0.001	0.002	< 0.001	< 0.001	< 0.001
SSc	0.486 (0.240)	0.583 (0.218)	0.580 (0.285)	0.736 (0.234)	< 0.001	< 0.001	< 0.001	< 0.001	< 0.001	0.770
OP	0.519 (0.242)	0.603 (0.233)	0.580 (0.319)	0.729 (0.258)	< 0.001	< 0.001	< 0.001	< 0.001	< 0.001	0.002
Diseases of the nervous system										
Epilepsy	0.804 (0.229)	0.826 (0.210)	0.831 (0.244)	0.900 (0.166)	0.001	< 0.001	0.006	< 0.001	< 0.001	0.384
MS	0.586 (0.252)	0.670 (0.222)	0.669 (0.278)	0.795 (0.195)	< 0.001	< 0.001	< 0.001	< 0.001	< 0.001	0.870
PD	0.476 (0.240)	0.583 (0.226)	0.588 (0.281)	0.741 (0.202)	< 0.001	< 0.001	< 0.001	< 0.001	< 0.001	0.523
Mental, behavioural and neurodevelopmental disorders										
Dementia	0.381 (0.288)	0.424 (0.286)	0.333 (0.430)	0.523 (0.405)	< 0.001	< 0.001	0.023	< 0.001	< 0.001	< 0.001
ADHD	0.697 (0.188)	0.727 (0.175)	0.735 (0.222)	0.846 (0.142)	< 0.001	< 0.001	0.005	< 0.001	< 0.001	0.320
SCZ	0.626 (0.214)	0.658 (0.212)	0.644 (0.295)	0.778 (0.227)	0.002	< 0.001	0.320	< 0.001	< 0.001	0.261
Diseases of the genitourinary system										
ENDO	0.880 (0.146)	0.888 (0.136)	0.902 (0.124)	0.950 (0.066)	0.001	< 0.001	< 0.001	< 0.001	< 0.001	< 0.001
BPH	0.792 (0.228)	0.838 (0.181)	0.852 (0.187)	0.913 (0.114)	< 0.001	< 0.001	< 0.001	< 0.001	< 0.001	< 0.001
OAB	0.611 (0.256)	0.678 (0.227)	0.668 (0.314)	0.787 (0.253)	< 0.001	< 0.001	0.005	< 0.001	< 0.001	0.489
Diseases of the skin and subcutaneous tissue										
PSO	0.647 (0.271)	0.706 (0.246)	0.694 (0.310)	0.808 (0.226)	< 0.001	< 0.001	< 0.001	< 0.001	< 0.001	0.056
Neoplasms										
BC	0.729 (0.236)	0.775 (0.205)	0.784 (0.242)	0.874 (0.152)	< 0.001	< 0.001	< 0.001	< 0.001	< 0.001	0.051
Endocrine, nutritional and metabolic diseases										
DM	0.665 (0.276)	0.728 (0.243)	0.723 (0.295)	0.826 (0.220)	< 0.001	< 0.001	< 0.001	< 0.001	< 0.001	0.350
Diseases of the circulatory system										
PAOD	0.413 (0.252)	0.508 (0.274)	0.426 (0.411)	0.589 (0.359)	< 0.001	< 0.001	0.527	< 0.001	< 0.001	< 0.001
Diseases of the eye and adnexa										
AMD	0.622 (0.262)	0.679 (0.250)	0.657 (0.334)	0.780 (0.246)	< 0.001	< 0.001	0.013	< 0.001	< 0.001	0.020
Total^a^	0.598 (0.279)	0.661 (0.257)	0.644 (0.334)	0.770 (0.261)	< 0.001	< 0.001	< 0.001	< 0.001	< 0.001	< 0.001

*ADHD* attention-deficit/hyperactivity disorder, *AMD* age-related macular degeneration, *BC* bladder cancer, *BPH* benign prostatic hyperplasia, *DM* diabetes mellitus, *ENDO* endometriosis, *EU* European, *NA* not available, *MS* multiple sclerosis, *OAB* overactive bladder, *OP* osteoporosis, *PAOD* peripheral arterial occlusive disease, *PL* polish, *PsA* psoriatic arthritis, *PSO* psoriasis, *RA* rheumatoid arthritis, *SCZ* schizophrenia, *SI* Slovenian, *SSc* systemic sclerosis, *UK* British

^a^Weighted mean and percentage values using analytical weights summing up to 100 in each disease

### Comparison of value sets by patients’ subjective health assessment

**Fig. 3:**
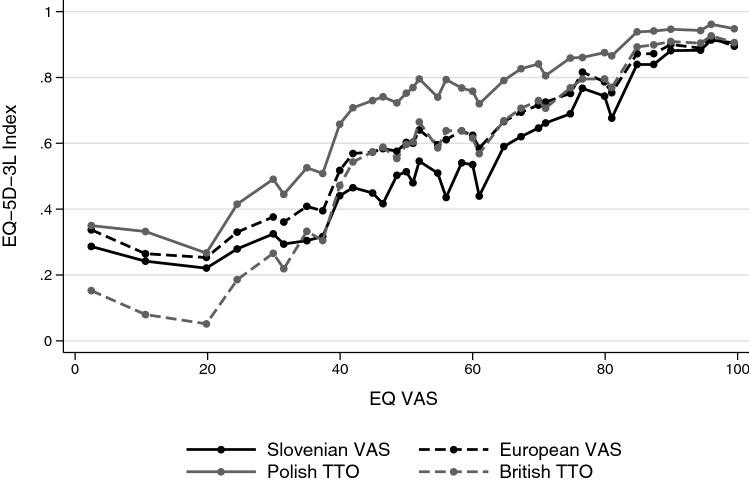
Comparison of value sets by patients’ subjective health assessment. The dots indicate 36 EQ VAS quantiles

We explored how disease severity influenced the differences between value sets. As a proxy for disease severity, we used subjective health assessments by the EQ VAS. We observed three distinct EQ VAS regions based on the pattern of value set differences (Fig. 3). Across the entire EQ VAS range, the Polish index values were highest. We observed greatest differences between the four value sets in the EQ VAS range between 40 and 80 (*n* = 437, 61.7%). In this range, the Slovenian index values were lowest, while the European and British value sets provided nearly identical index values. Differences were the smallest in the EQ VAS range between 80 and 100 (*n* = 437, 20.4%), and the pattern of value sets changed below EQ VAS levels of 40 (*n* = 437, 17.9%), where the Slovenian, European and Polish value sets converged, and the British value set provided the lowest index values.

### Comparison of value sets by age groups

We aimed to analyse whether the EQ-5D-3L index scores calculated with the four value sets differed depending on the patients’ age. Three age groups were created: 18–34, 35–54 and 55+ years old. From the 2421 patients 16.3% belonged to the youngest (*n* = 275), 26.7% to the middle (*n* = 609) and 56.6% (*n* = 1525) to the oldest age group.

More than half of the patients belonged to the youngest age group in ADHD (69.3%), endometriosis (64.6%) and epilepsy (54.2%). In certain diseases that typically affect the elderly, most of the patients were over 55 years old. In AMD 100%, in dementia 96.5%, in BPH 95.8%, in PAOD 95.2% and in OP 93.7% of the patients belonged to the oldest age group.

The Slovenian, European, Polish and British index values were 0.765 (SD = 0.214), 0.793 (SD = 0.190), 0.886 (SD = 0.140) and 0.804 (SD = 0.213) in the 18–34 age group, 0.601 (SD = 0.277), 0.662 (SD = 0.255), 0. 773 (SD = 0.251) and 0.644 (SD = 0.329) in the 35–54 age group, and 0.548 (SD = 0.277), 0.622 (SD = 0.262), 0.735 (SD = 0.281) and 0.598 (SD = 0.350) in the 55+ age group, respectively. All value set comparisons differed significantly (*p *≤ 0.001). In all age groups, index values were lowest when measured with the Slovenian, followed by the European and the British value sets, while the Polish index values were highest. The difference between index values of the youngest and eldest age groups was biggest when using the Slovenian (0.217), followed by the British (0.206), the European (0.171) and the Polish (0.150) value sets. All age group comparisons were significant (*p* < 0.001).

### The sensitivity of disease burden evaluation to the choice of the value set

**Fig. 4:**
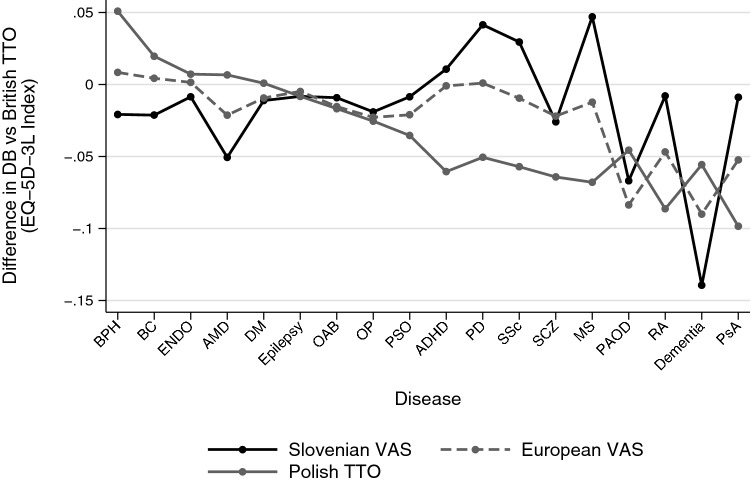
Differences of DB evaluations compared to the British TTO value set. Conditions are ordered from left to right in increasing disease burden according to the British value set. *ADHD* attention deficit hyperactivity disorder, *AMD* age-related macular degeneration, *BC* bladder cancer, *BPH* benign prostatic hyperplasia, *DM* diabetes mellitus, *ENDO* endometriosis, *MS* multiple sclerosis, *OAB* overactive bladder, *OP* osteoporosis, *PAOD* peripheral arterial occlusive disease, *PsA* psoriatic arthritis, *PSO* psoriasis, *RA* rheumatoid arthritis, *SCZ* schizophrenia, *SSc* systemic sclerosis

Although the British TTO value set is used most frequently for the evaluation of health outcomes in Hungary as well as the CEE region [8, 51], we explored how DB evaluations would be affected in the 18 conditions by choosing a different value set (Fig. 4). We observed differences in both positive and negative directions in certain conditions with all value sets, with most striking differences between the British and Slovenian ones. The DB score of dementia was nearly 0.15 index points lower, while for MS and PD nearly 0.05 index points higher when choosing the Slovenian value set instead of the British one. The Polish value set differed most from the British one in PsA, while the European one in dementia. The difference was also substantial between the Polish and Slovenian value sets in MS and PD as well. The value sets performed nearly identically in DM, epilepsy, OAB and OP. The difference from the British value set tended to increase with the level of disease burden in all value sets.

As a general measure of sensitivity to the choice of the value set, we introduced the sensitivity index (Table 3). Altogether, from the 153 pairwise comparisons of DB scores that were evaluated between the 18 conditions (*n* = 18*17/2), 22.9% provided diverse outcomes. Most of the diverse outcomes were combinations of non-significant differences and significant differences in one direction. Mainly due to the modest sample sizes of the disease subsamples, there were no pairwise comparisons where alternative value sets provided statistically significant reverse DB rankings. (+ ΔDB_*ij*_ for one value set, but—for another.) The sensitivity index suggested that the disease conditions are not identically sensitive to the choice of the value set. The outcome of DB comparisons including conditions such as dementia, MS or PAOD depended strongly on the value set, while DB comparisons of other diseases, such as BPH and BC, were hardly affected. The sensitivity index also provided information about how the choice between two value sets influenced DB evaluation results across the 18 conditions. Overall, the Slovenian and Polish value sets provided the most divergent DB comparisons: in 21.2% of the DB comparisons, the choice between the Slovenian and Polish value sets provided a divergent outcome. At the other extreme, the choice between the European and Slovenian value sets hardly affected the outcome of DB comparisons, just as the choice between the Polish and the British value sets.

**Table 3:** Sensitivity of DB comparisons to the choice of value set (sensitivity index)

	Number of comparisons	Proportion of inconsistent pairwise DB differences based on comparing						
		All four value sets^a^	EU vs. PL value set^c^	EU vs. SI value set^c^	EU vs. UK value set^c^	SI vs. PL value set^c^	SI vs. UK value set^c^	PL vs. UK value set^c^
Diseases of the musculoskeletal system and connective tissue								
RA	17	0.118	0.059	0.000	0.059	0.118	0.118	0.000
PsA	17	0.176	0.118	0.000	0.118	0.118	0.118	0.000
SSc	17	0.294	0.294	0.000	0.176	0.294	0.176	0.118
OP	17	0.235	0.235	0.000	0.118	0.235	0.118	0.118
Diseases of the nervous system								
Epilepsy	17	0.118	0.059	0.000	0.000	0.059	0.118	0.059
MS	17	0.412	0.235	0.059	0.059	0.412	0.235	0.176
PD	17	0.294	0.294	0.000	0.294	0.294	0.294	0.000
Mental, behavioural and neurodevelopmental disorders								
Dementia	17	0.412	0.235	0.118	0.235	0.412	0.412	0.000
ADHD	17	0.294	0.294	0.000	0.294	0.294	0.294	0.000
SCZ	17	0.294	0.294	0.000	0.118	0.235	0.059	0.176
Diseases of the genitourinary system								
ENDO	17	0.176	0.118	0.059	0.000	0.118	0.118	0.118
BPH	17	0.000	0.000	0.000	0.000	0.000	0.000	0.000
OAB	17	0.294	0.294	0.000	0.235	0.294	0.235	0.059
Diseases of the skin and subcutaneous tissue								
PSO	17	0.294	0.235	0.118	0.235	0.235	0.235	0.000
Neoplasms								
BC	17	0.059	0.000	0.059	0.000	0.059	0.059	0.000
Endocrine, nutritional and metabolic diseases								
DM	17	0.118	0.059	0.000	0.000	0.118	0.059	0.059
Diseases of the circulatory system								
PAOD	17	0.353	0.353	0.000	0.235	0.353	0.235	0.118
Diseases of the eye and adnexa								
AMD	17	0.176	0.059	0.118	0.059	0.176	0.176	0.000
Total	153	0.229^b^	0.180^d^	0.029^d^	0.124^d^	0.212^d^	0.170^d^	0.056^d^

*ADHD* attention-deficit/hyperactivity disorder, *AMD* age-related macular degeneration, *BC* bladder cancer, *BPH* benign prostatic hyperplasia, *DM* diabetes mellitus, *ENDO* endometriosis, *EU* European, *NA* not available, *MS* multiple sclerosis, *OAB* overactive bladder, *OP* osteoporosis, *PAOD* peripheral arterial occlusive disease, *PL* polish, *PsA* psoriatic arthritis, *PSO* psoriasis, *RA* rheumatoid arthritis, *SCZ* schizophrenia, *SI* Slovenian, *SSc* systemic sclerosis, *UK* British

^a^Calculated from 17×4 DB evaluations

^b^Calculated from 153×4 DB evaluations

^c^Calculated from 17×2 DB evaluations

^d^Calculated from 153×2 DB evaluations

## Discussion

We analysed the systematic differences between four value sets and their potential impact on health priority setting across 18 chronic conditions [8, 31–48]. We chose value sets that are mostly used or potentially applicable to health economic evaluations in the CEE region [8]. Our analysis was conducted on a broad range of immune-mediated inflammatory diseases, neurological, mental, urological and other disorders from cross-sectional samples recruited in Hungary [8, 31–48]. We compared patient-level utility values using the four value sets in each condition with individual-level data from a population-norm for the CEE region. Former studies compared different TTO and VAS-based EQ-5D-3L value sets among the general populations of different countries [11], TTO-based value sets from different econometric models on a single patient population [12, 52], three TTO-based value sets in a single general population [53] and composite TTO (cTTO) and discrete-choice experiment (DCE) based value sets from six countries across seven patient populations [54]. Numerous studies compared value sets by each EQ-5D-3L profile [13, 29, 55]. Our study is unique in terms of comparing value sets derived by different methods (VAS and TTO) from populations with different cultural and economic background (CEE and Western Europe) across multiple patient populations. Although the 18 conditions were chosen arbitrarily, the breadth of disease areas, as well as the severity of conditions, allowed for a unique systematic comparison of disease burden estimates that allows the generalisation of our results beyond the boundaries of the Hungarian healthcare system.

We found remarkable differences across diagnoses, age groups or patients with different disease severity. For example, the mean EQ-5D-3L index difference was as high as 0.265 in PD and 0.187 in the 55 + age group. The systematic pairwise comparison of DB evaluations between all disease conditions revealed inconsistent results between value sets determined via the VAS and TTO valuation methodology. The proportion of discrepant DB evaluations was 21.9% when comparing the Slovenian VAS and Polish TTO value sets. However, value sets determined via the same methodology provided rather consistent results. Only 2.9% of DB evaluations were discrepant when comparing the VAS-based Slovenian and European value sets, and 5.6% when comparing the TTO-based Polish and British value sets, despite the apparent cultural or economic differences between the populations whose preferences were valued. Our results confirm the findings of previous studies highlighting the methodology-driven differences between EQ-5D-3L value sets [11, 54].

The differences between EQ-5D-3L value sets can lead to significant differences in health gains in EQ-5D-3L-based cost-utility analyses. Taking the extremes, for example, if the health status of a patient is improved from ‘22222’ (moderate problems in all dimensions) to ‘11111’ (perfect health) over a year with a new treatment, the QALY gain will be 0.685 with the Slovenian, but only 0.284 with the Polish value set. Improvement from the worst possible health state (‘33333’) to a moderate health state (‘22222’) would result in 1.239 QALY gain with the Polish, but only 0.555 with the European value set. Results of the DB evaluations and the examples for the potential QALY-gain differences pinpoint that the value set chosen to calculate health state utilities in chronic diseases might significantly influence the results as well as the health policy and financing decisions. These findings deserve careful consideration in health economic analyses in the CEE, where local data are sparse, and economic analyses frequently need to rely on a variety of external sources. [56].

There is increasing interest in developing country-specific value sets. Local value sets could reflect better the health preferences of a given population (people’s beliefs about how particular problems in the EQ-5D dimensions impact their health-related quality of life) than value sets derived in a foreign country [57]. By feeding into further analyses, major utility score differences owing to the choice of value set can contribute to disparities in health policy and financing decisions due to over- or underestimation of treatment outcomes or different prioritisation of diseases. Although populations’ preferences might be reflected better by country-specific value sets, international comparisons of access differences, for example, are getting difficult to interpret and less useful for policymaking as it may be difficult to disentangle disparities between countries driven by different evaluations of disease burden or treatment outcomes, or other factors related to the local healthcare system. Hence, using a single value set, adjusted [58] or even unweighted scores [59, 60] would aid cross-country comparisons by diminishing the effect of differences arising from different methodologies or health preferences.

The differences of the value sets might at least partly explain the differences between Poland and Hungary in terms of access to highly effective but costly biological drugs in chronic immune-mediated diseases. Several studies were conducted involving these two countries in CEE to evaluate access to biologicals in rheumatoid arthritis, psoriatic arthritis, ankylosing spondylitis, Crohn’s disease, ulcerative colitis and psoriasis. [33, 61–63]. Although the access to biologicals correlated strongly with GDP per capita among European countries [64], substantial differences were found in the uptake among countries with similar economic development such as Poland and Hungary [33]. Access to biologicals varies widely among CEE countries, and this difference could not be explained by epidemiological factors, drug prices or total health expenditure.

Variations of reimbursement policy may be one of the factors explaining the differences to a certain extent in Bulgaria, Latvia, Lithuania, and Poland, but the association with other possible.

Determinants (differences in prevalence and incidence, the price of biologicals, total expenditure on health, geographical access, and cost-effectiveness results) were not proven. We assumed, nevertheless, in these papers that health deterioration linked to these diseases might be valued differently against other systemic inflammatory conditions in distinct countries and which may contribute to the immense diversity in the utilisation of biological drugs for immune-mediated chronic diseases. However, comparison of Hungary and Poland which have very similar total health expenditure refutes this assumption since in Hungary the exposure to biologicals used to be approximately ten times higher compared to Poland in inflammatory bowel diseases [51], despite the chronic financial deficits of the Hungarian healthcare system [65]. Similar differences were seen using biologics in psoriasis [63] and rheumatoid disorders [62, 66].

In addition to the availability of patient-level data from 18 conditions, a unique strength of our study is that we could calculate disease burden scores with the four value sets using population norm database involving patient-level data of 6926 respondents. Our database contained 997 individuals in the 65+ age group, allowing for potentially more reliable DB estimates in older populations than the available local population norm values [4, 5]. However, our analysis has some limitations. The patients included in our sample may not be representative of the entire patient population in the given diagnosis. Further research including other diseases could help to understand in condition-specific differences that can be detected in the EQ-5D-3L index values calculated with different value sets. Comparison with other value sets could aid a better understanding of the factors influencing the differences between national value sets coming from different regions.

As a conclusion, based on the analysis of a wide range of chronic conditions and a variety of value sets in terms of the population and method of valuation, our study highlights the potential impact of value set choice on health policy decisions.
